# Microsatellite Stable Colorectal Cancers Stratified by the *BRAF* V600E Mutation Show Distinct Patterns of Chromosomal Instability

**DOI:** 10.1371/journal.pone.0091739

**Published:** 2014-03-20

**Authors:** Catherine E. Bond, Derek J. Nancarrow, Leesa F. Wockner, Leanne Wallace, Grant W. Montgomery, Barbara A. Leggett, Vicki L. J. Whitehall

**Affiliations:** 1 Conjoint Gastroenterology Laboratory, QIMR Berghofer Medical Research Institute, Brisbane, Queensland, Australia; 2 School of Medicine, University of Queensland, Brisbane, Queensland, Australia; 3 Cancer Control Group, QIMR Berghofer Medical Research Institute, Brisbane, Queensland, Australia; 4 Oncogenomics Group, QIMR Berghofer Medical Research Institute, Brisbane, Queensland, Australia; 5 Cancer and Population Studies Group, QIMR Berghofer Medical Research Institute, Brisbane, Queensland, Australia; 6 Molecular Epidemiology Group, QIMR Berghofer Medical Research Institute, Brisbane, Queensland, Australia; 7 Royal Brisbane and Women's Hospital, Brisbane, Queensland, Australia; 8 Pathology Queensland, Brisbane, Queensland, Australia; University Hospital Carl Gustav Carus, Germany

## Abstract

The *BRAF* (V600E) mutation in colorectal cancers that are microsatellite stable (MSS) confers a poor patient prognosis, whereas *BRAF* mutant microsatellite-unstable (MSI) colorectal cancers have an excellent prognosis. *BRAF* wild type cancers are typically MSS and display chromosomal instability (CIN). CIN has not been extensively studied on a genome-wide basis in relation to *BRAF* mutational status in colorectal cancer. *BRAF* mutant/MSS (*BRAF*mut/MSS) cancers (n = 33) and *BRAF* mutant/MSI (*BRAF*mut/MSI) cancers (n = 30) were compared for presence of copy number aberrations (CNAs) indicative of CIN, with *BRAF* wild type/MSS (*BRAF*wt/MSS) cancers (n = 18) using Illumina CytoSNP-12 arrays. *BRAF*mut/MSS and *BRAF*wt/MSS cancers showed comparable numbers of CNAs/cancer at 32.8 and 29.8 respectively. However, there were differences in patterns of CNA length between MSS cohorts, with *BRAF*mut/MSS cancers having significantly greater proportions of focal CNAs compared to *BRAF*wt/MSS cancers (p<0.0001); whereas whole chromosomal arm CNAs were more common in *BRAF*wt/MSS cancers (p<0.0001). This related to a reduced average CNA length in *BRAF*mut/MSS compared to *BRAF*wt/MSS cancers (20.7 Mb vs 33.4 Mb;p<0.0001); and a smaller average percent of CIN affected genomes in *BRAF*mut/MSS compared to *BRAF*wt/MSS cancers (23.9% vs 34.9% respectively). *BRAF*mut/MSI cancers were confirmed to have low CNA rates (5.4/cancer) and minimal CIN-affected genomes (average of 4.5%) compared to MSS cohorts (p<0.0001). *BRAF*mut/MSS cancers had more frequent deletion CNAs compared to *BRAF*wt/MSS cancers on 6p and 17q at loci not typically correlated with colorectal cancer, and greater amplification CNAs on 8q and 18q compared to *BRAF*wt/MSS cancers. These results indicate that comparable rates of CIN occur between MSS subgroups, however significant differences in their patterns of instability exist, with *BRAF*mut/MSS cancers showing a ‘focal pattern’ and *BRAF*wt/MSS cancers having a ‘whole arm pattern’ of CIN. This and the genomic loci more frequently affected in *BRAF*mut/MSS cancers provides further evidence of the biological distinctions of this important cancer subgroup.

## Introduction

The *BRAF* V600E mutation is present in approximately 10–15% of sporadic colorectal cancer (CRC) [1] and is a hallmark of the serrated neoplastic pathway of CRC, where cancers develop from serrated precursor polyps [2], [3]. The CpG Island Methylator Phenotype (CIMP) is strongly associated with presence of the *BRAF* mutation [2], [4], [5]. In approximately half of these *BRAF* mutant cancers, CIMP related methylation and silencing of the DNA mismatch repair gene, *MLH1*, results in widespread frameshift mutations known as microsatellite instability (MSI). *BRAF* mutant/MSI cancers have been well characterized and show typical molecular and clinical features including an excellent patient outcome [4], [6], [7], [8], [9]. The remaining *BRAF* mutant cancers do not methylate *MLH1* and are microsatellite stable (MSS). These *BRAF* mutant/MSS cancers have not been as well studied, but importantly confer a very poor patient prognosis [9], [10], [11].

The majority of sporadic CRC are *BRAF* wild type and arise from conventional adenomas that follow a well defined pathway of molecular events leading to cancer [12]. These *BRAF* wild type cancers are typically MSS and frequently show chromosomal instability (CIN) [8], the presence of which has been correlated with a poor prognosis in these cancers [13], [14], [15], [16]. Interestingly, the presence of the *BRAF* V600E mutation in MSS cancers confers an even worse prognosis [9], [17], however CIN has not been extensively studied on a genome-wide basis in this cancer subgroup.

CIN refers to the rate of acquisition of copy number aberrations (CNAs) where sections of DNA are affected by either deletion or amplification events [18]. CIN can affect whole chromosomes largely through dysfunctional chromosome segregation during mitosis [18], [19], and aneuploidy is the stable state of abnormal chromosome numbers [18]. Alternatively CIN can refer to the presence of widespread structural sub-chromosomal rearrangements resulting from incorrect repair of DNA damage [20]. These structural rearrangements can arise though repetitive rounds of breakage and fusion repair cycles leading to complex deletions, amplifications and translocations [21].

Few studies have extensively investigated CIN in the context of *BRAF* mutational and MSI status. We have previously found comparably high frequencies of LOH events between *BRAF* mutant/MSS cancers and *BRAF* wild type cancers at several key genomic loci (18q, 17p, 5q and 8p), that are known to harbour important tumour suppressor genes [22].

Application of genome-wide single nucleotide polymorphism (SNP) arrays to study the presence of CIN has allowed the identification of different types of CNAs including complex aberrations and copy neutral loss of heterozygosity (cnLOH) events. Several common regions targeted by CNAs in CRC including deletions on chromosomes 17p, 18q, 5q, 8p, 4q and 1p, and amplifications on chromosomes 13q, 20q, 7p, 7q and 8q, have been confirmed through SNP array studies [23], [24].

MSI and CIN have previously been considered as two distinct pathways of genomic instability due to findings of MSI cancers being largely diploid [13], [25], [26]. However, several studies using cytogenetic analysis have found MSI cell lines and cancers to have a considerable presence of chromosomal aberrations, predominantly cnLOH events [27], [28], [29], [30]. Similarly, studies have reported the presence of CIN and CIMP to be inversely correlated [31], [32], and the incidence of frequent methylation to be associated with reduced rates and lengths of CNAs [33], [34], [35]. However, the majority of these studies did not stratify for presence of a *BRAF* mutation [31], [33], [35].

We and others have highlighted the importance of the *BRAF*mut/MSS cancer type with their correlations with poor patient outcomes and presence of distinct molecular and clinical features [9], [10], [17], [22], [36]. This study expands on the characterization of these cancers by investigating the extent of CIN on a genome-wide basis which may help to determine further molecular aberrations that could be contributing to the aggressiveness of this cancer type.

## Materials and Methods

### Cancer Samples

An initial cohort of 1052 sporadic colorectal cancers and matched normals were obtained from patients following surgery at the Royal Brisbane and Women's Hospital, Queensland, Australia. Written, informed consent was collected from all patients, and the study was approved under the RBWH and Bancroft Human Research Ethics Committee. Clinical data including patient gender, age, stage at diagnosis (American Joint Committee on Cancer, AJCC), and anatomical site of cancer (with proximal location considered as being proximal to the splenic flexure) was collected where available.

*BRAF, p53 and KRAS Mutation, MSI and CIMP Investigations*: All cancer samples had previously been investigated for MSI status using the 5 marker panel of the National Cancer Institute (mononucleotide: BAT25, BAT26; dinucleotide: D5S346, D2S123, D17S250) and classified MSI if at least two markers, including at least one mononucleotide marker, were positive. The presence of the *BRAF* V600E mutation, *p53* mutation (across exons 4 to 8), *KRAS* mutation (at codons 12 and 13), and the CpG Island Methylator Phenotype (using a 5 marker panel consisting of *CACNA1G*, *IGF2*, *NEUROG1*, *RUNX3*, *SOCS1* [5]) had also been previously determined [22], [36], [37]. The cancers were subsequently divided into three cohorts depending on their MSI and *BRAF* mutational status: as *BRAF* mutant/MSS (n = 60), *BRAF* mutant/MSI (n = 68) or *BRAF* wild type/MSS (n = 924).

### Single Nucleotide Polymorphism Arrays

From these cohorts, 33 *BRAF* mutant/MSS, 30 *BRAF* mutant/MSI and 18 *BRAF* wild type/MSS cancers and matched normal samples were chosen for quantification using Picogreen dye, and analysis for genome-wide copy number aberrations (CNAs) with HumanCytoSNP-12v2.1 Single Nucleotide Polymorphism (SNP) arrays (Illumina; San Diego, Ca.) according to the manufacturer's instructions. The beadchips were scanned using Illumina's iScan system and the image data was analysed with Illumina's GenomeStudio version 2011.1.0.24550. The cancer traces were referenced to their matched normal profiles and the boundaries of all somatic copy number aberrations were manually determined and based on human genome build NCBI36/hg19. To account for stromal contamination commonly present in cancer samples, the Simulated DNA Copy Number (SiDCoN) [38] and an automated SiDCoN2 tool which are R script based applications, were used to assign each CNA a log R ratio and B allele frequency score to determine the genotype of each CNA. The SiDCoN application was able to determine the extent of cells that showed an aberrant copy number change for each CNA, including heterogenous genotypes. Any individual CNA that scored less than 20% of aberrant cellular involvement was excluded from analysis in order to ensure reliable CNA data [38], [39]. The CNAs were then converted from Excel to custom data tracks and visualized on the University of California, Santa Cruz's Genome Browser (http://genome.ucsc.edu/) [40].

Cytogenetics terminology was applied with ‘gains’ and ‘losses’ referring to whole chromosome arm events where a large genomic region was affected and typically consisted of small copy numbers. ‘Amplifications’ and ‘deletions’ referred to CNAs covering sub-chromosomal or focal regions and these potentially involved greater copy numbers [41]. Specific types of deletion and amplification CNAs were analysed with deletion events comprising of loss of heterozygosity (LOH), copy neutral loss of heterozygosity (cnLOH) and homozogous deletion (HD) events, whilst amplification CNAs included 3n and ≥4n (complex) amplification events.

To identify the extent of CNA coverage per chromosome, the length of each CNA was calculated as a fraction of its coverage over the full length of the specific chromosome arm in order to allow for comparisons of CNAs occurring on all chromosome arms with differing lengths [41]. Continuous CNAs covering ≥95% of a chromosomal arm were termed ‘whole chromosome arm’ CNAs; regional CNAs covered between 50–94% of a chromosome arm; and focal events were considered as <50% the length of a chromosome arm in keeping with previously published data [24], [41], [42], [43]. In this study, whole chromosome CNAs (continuous aberrations extending over both chromosome arms) were included in the analysis of whole chromosome arm CNAs as in Beroukhim *et al* [41] and the Cancer Genome Atlas Network's characterization of CRC [24]. Minimal common regions (MCRs) were also identified and referred to the smallest genomic loci that contained deletion or amplification copy number changes at the highest frequencies across cancers in each cohort.

*Cancer Cell Density in Samples*: SiDCoN assisted in estimating the cancer cell density of each sample [39], and those samples that contained >40% of tumour cells were automatically included for analysis (Figure S1 in File S1). As described by Dulak et al [43], the remaining cancers (*BRAF* mutant/MSS 11/33 = 33%; *BRAF* mutant/MSI 12/30 = 40%; *BRAF* wild type/MSS 2/18 = 11%) were analysed for presence of co-existing molecular changes relating to tumourigenesis in order to confirm there was a sufficient ratio of tumour cell compared to normal cell content to justify molecular analyses. This analysis included the presence of methylated markers, evidence of MSI and LOH, and mutations of cancer-related genes [22], [36] (Table S1 in File S1). Data and statistical differences with these cancer samples either excluded or not were compared to verify their inclusion in this study (Table S2 in File S1).

### Statistical Analysis

Significant differences between categorical data were analysed with Pearson's chi-squared test, or Fisher's exact test where appropriate. Proportions were tested using a proportion test, and where appropriate these p-values were corrected for multiple comparisons using the Benjamini-Hochberg method. For continuous variables, ANOVA was used to test for a significant difference between groups, and Post-Hoc analysis (using Tukey's HSD) was performed to explore differences further. For tests within cohorts, either a paired t-test or Wilcox's sign rank test and Friedman's test of related samples was performed. P values ≤0.05 were considered significant.

## Results

### Clinical and Molecular Features of Study Cohorts

33 *BRAF* mutant/MSS (*BRAF*mut/MSS), 18 *BRAF* wild type/MSS (*BRAF*wt/MSS), and 30 *BRAF* mutant/MSI (*BRAF*mut/MSI) cancers were analysed. The majority of *BRAF* mutant cancers derived from the proximal colon, whereas most *BRAF*wt/MSS cancers were found distally (p<0.0001) (Table 1). The *BRAF*mut/MSS cancers presented mostly at advanced stages (AJCC III and IV), compared to *BRAF*wt/MSS and *BRAF*mut/MSI cancers (p = 0.03) (Table 1). *BRAF*mut/MSI cancers conferred a later age of onset compared to MSS cancers (p = 0.01. Molecularly, the CpG Island Phenotype (CIMP) was predominant in the *BRAF* mutant cohorts, particularly the *BRAF*mut/MSI cancers; whereas no *BRAF*wt/MSS cancers were CIMP high (p<0.0001) (Table 1). *KRAS* mutations were present in 28% *BRAF*wt/MSS cancers and the mutual exclusivity of *KRAS* with *BRAF* mutations was confirmed.

**Table 1:** Clinical and molecular data of cohorts.

Feature	*BRAF*mut/MSS	*BRAF*wt/MSS	*BRAF*mut/MSI	P Value
**n**	33	18	30	
**Average Age at Onset**	68.5	69.1	76.2	**0.01**
**Female Gender**	21/33 (64%)	8/18 (44%)	22/30 (73%)	0.13
**Proximal Location**	21/31 (70%)	4/18 (22%)	27/29 (93%)	**<0.0001**
**AJCC Stage I**	1/25 (4%)	3/18 (17%)	7/27 (26%)	**0.03**
**AJCC Stage II**	9/25 (36%)	8/18 (44%)	16/27 (59%)	
**AJCC Stage III**	10/25 (40%)	5/18 (28%)	2/27 (7%)	
**AJCC Stage IV**	5/25 (20%)	2/18 (11%)	2/27 (7%)	
**CIMP High**	17/30 (57%)	0/18	21/30 (70%)	**<0.0001**
***p53*** ** Mutant**	12/30 (40%)	9/18 (50%)	7/30 (23%)	0.17
***KRAS*** ** Mutant**	0	5/18 (28%)	0	-

### Rates of Copy Number Aberrations in Molecular Subgroups

Individual copy number aberrations (CNAs) that had ≥20% cellular involvement as determined by SiDCoN [38], [39] were included in the following analysis. Cancers that had less than 40% tumour content as estimated by SiDCoN [39] had substantial evidence of cancer related molecular changes (Table S1 in File S1) [43], suggesting sufficient tumour cellularity to also detect CNAs. Statistical differences in the rate and type of CNAs occurring between cohorts remained valid when analyses were performed with their exclusion (Table S2). Therefore the full cohorts were considered for this investigation.

The MSS cohorts had comparable average rates of CNAs per cancer with a rate of 32.8 per *BRAF*mut/MSS cancer and 29.8 per *BRAF*wt/MSS cancer. The *BRAF*mut/MSI cohort had a significantly lower rate of 5.4 CNAs per cancer (p<0.0001) (Table 2).

**Table 2:** Extent of CNAs per Cohort.

	*BRAF*mut/MSS	Paired t-test within BRAFmut/ MSS	*BRAF*wt/MSS	Paired t-test within BRAF wt/ MSS	P Value between MSS Cohorts *	*BRAF*mut/MSI	P Value between all 3 cohorts
**n**	33	-	18	-	-	30	-
**Total Number of CNAs in Cohort**	1084	-	536	-	-	162	-
**Average Number CNAs per Cancer**	32.8	-	29.8	-	0.86	5.4	**<0.0001**
**Average Length of CNA (Mb)**	20.7	-	33.4	-	**<0.0001**	23.6	**<0.0001**
**Median Fraction of CNA over chromosome arm lengths**	0.09	-	0.39	-	**<0.0001**	0.02	**<0.0001**
**Av. Fraction of CNA over chromosome arm lengths**	0.32	-	0.50	-	**<0.0001**	0.36	**<0.0001**
**Deletion CNAs in Cohort**	815/1084 (75.2%)	-	372/536 (69.4%)	-	**0.016**	120/162 (74.1%)	**0.045**
**Amplification CNAs in Cohort**	269/1084 (24.8%)	-	164/536 (30.6%)	-	-	42/162 (25.9%)	**-**
**Average No. Deletion CNAs per cancer**	24.7	**<0.0001**	20.7	**0.0006**	0.65	4.0	**<0.0001**
**Average No. Amplification CNAs per cancer**	8.2		9.1		0.92	1.4	**0.0005**
**Av. % of Genome Affected by CNAs per cancer**	23.9%	-	34.9%	-	0.1	4.5%	**<0.0001**
**Av % of Genome Affected by Deletion CNAs per cancer**	18.6%	**<0.0001**	22.7%	0.018	0.56	2.2%	**<0.0001**
**Av % of Genome Affected by Amplification CNAs per cancer**	5.2%		12.2%		**0.012**	2.2%	**<0.0001**
**Whole Chromosome Arm CNAs in Cohort**	187/1084 (17.3%)	-	171/536 (31.9%)	-	**<0.0001**	41/162 (25.3%)	**<0.0001**
**Regional CNAs in Cohort**	131/1084 (12.1%)	-	71/536 (13.2%)	-	0.5	18/162 (11.1%)	0.70
**Focal CNAs in Cohort**	766/1084 (70.7%)	-	294/536 (54.9%)	-	**<0.0001**	103/162 (63.6%)	**<0.0001**
**Average No. Whole Arm CNAs per cancer**	5.7	**<0.0001**	9.5	**0.0091**	**0.04**	1.4	**<0.0001**
**Average No. Regional CNAs per cancer**	4.0		3.9		0.99	0.6	**<0.0001**
**Average No. Focal CNAs per cancer**	23.2		16.3		0.35	3.4	**<0.0001**
**Average No. Whole Arm Loss CNAs per cancer**	4.8	**<0.0001**	7.1	**0.001**	0.16	0.67	**<0.0001**
**Average No. Whole Arm Gain CNAs per cancer**	0.9		2.4		**0.01**	0.7	**0.004**
**Av. No. Regional Deletion CNAs per cancer**	3.1	**<0.0001**	2.5	**<0.0001**	0.4	0.4	**<0.0001**
**Av. No. Regional Amplification CNAs per cancer**	0.8		1.4		0.2	0.2	**0.006**
**Av. No. Focal Deletion CNAs per cancer**	16.8	**0.004**	11.0	**0.03**	0.3	2.9	**0.0008**
**Av. No. Focal Amplification CNAs per cancer**	6.4		5.3		0.7	0.5	**0.001**
**Av. % Genome Affected by Whole Arm CNAs**	11.9%	0.094	22.1%	**0.0002**	**0.018**	3.0%	**<0.0001**
**Av. % Genome Affected by Regional CNAs**	6.9%		6.6%		0.98	1.2%	**0.0001**
**Av. % Genome Affected by Focal CNAs**	5.1%		6.3%		0.68	0.3%	**<0.0001**

* Adjusted for multiple comparisons.

The average length of a single CNA in the *BRAF*wt/MSS cohort (33.4 Mb) was significantly longer than the average CNA length in the *BRAF*mut/MSS cohort (20.7 Mb) (p<0.0001), and the *BRAF*mut/MSI cohort (23.6 Mb) (p<0.0001) (Table 2). The length of each CNA was considered as a fraction of the length of the specific chromosome arm [41]. This showed significant differences in the average and median chromosome fraction affected by CNAs occurring between cohorts, with the *BRAF*wt/MSS having the highest fraction of chromosome arm involvement compared to the *BRAF* mutant cohorts (p<0.0001) (Table 2). This difference in average CNA length corresponded to a greater average percentage of genomes affected by CNAs in the *BRAF*wt/MSS cohort (34.9%; range 0–80.5%), compared to the *BRAF*mut/MSS cohort (23.9%; range 0–68.6%). In comparison to MSS cancers, *BRAF*mut/MSI cancers had a minimal proportion of genome involvement (4.5%; range 0–25.8%) (p<0.0001) (Table 2). Due to this small extent of CNAs affecting *BRAF*mut/MSI cancers, the following results will mainly compare the two MSS cohorts.

Deletion and amplification CNAs were considered as a rate of the total number of CNAs occurring within that cohort, as well as the number of events occurring per cancer within each cohort. Across all cohorts, deletion CNAs were more common than amplification CNAs, with deletion events constituting approximately 73% of all CNAs per cohort (Table 2). The *BRAF*wt/MSS cohort had a significantly greater average percentage of the genome affected by amplification events than *BRAF*mut/MSS cancers (12.2% vs 5.2% respectively; p = 0.01) (Table 2).

**Figure 1:**
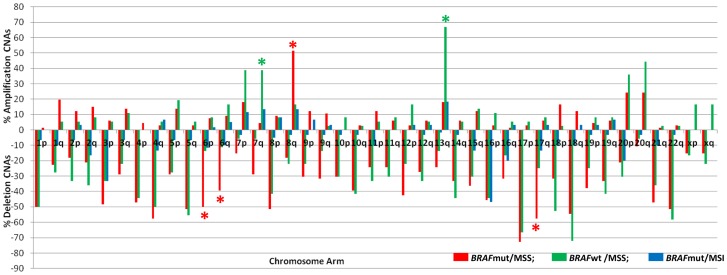
Percentage of cancers per cohort that had an amplification or deletion copy number aberration at each chromosome arm. Asterisks indicate those chromosome arms where significant differences (p<0.05) in the rate of CNAs per cancer occurred between MSS cohorts (red for the *BRAF*mut/MSS cohort and green for the *BRAF*wt/MSS cohort to indicate which has a significantly greater rate of CNAs per cancer).

The most frequent deletion events occurring in at least 50% of cancers in both MSS cohorts, involved chromosomes 1p, 4q, 5q, 17p, 18q and 22q. The *BRAF*mut/MSS cohort had significantly more common deletion events than the *BRAF*wt/MSS cohort at chromosomes 6p (p = 0.02), 6q (p<0.05) and 17q (p = 0.02) (Figure 1). Significantly more frequent amplification events occurred in *BRAF*wt/MSS compared to *BRAF*mut/MSS cancers at 13q (p = 0.0009) and 7q (p = 0.006). The *BRAF*mut/MSS cancers had significantly more frequent amplification events at 8q compared to *BRAF*wt/MSS cancers (p = 0.02) (Figure 1).

**Figure 2:**
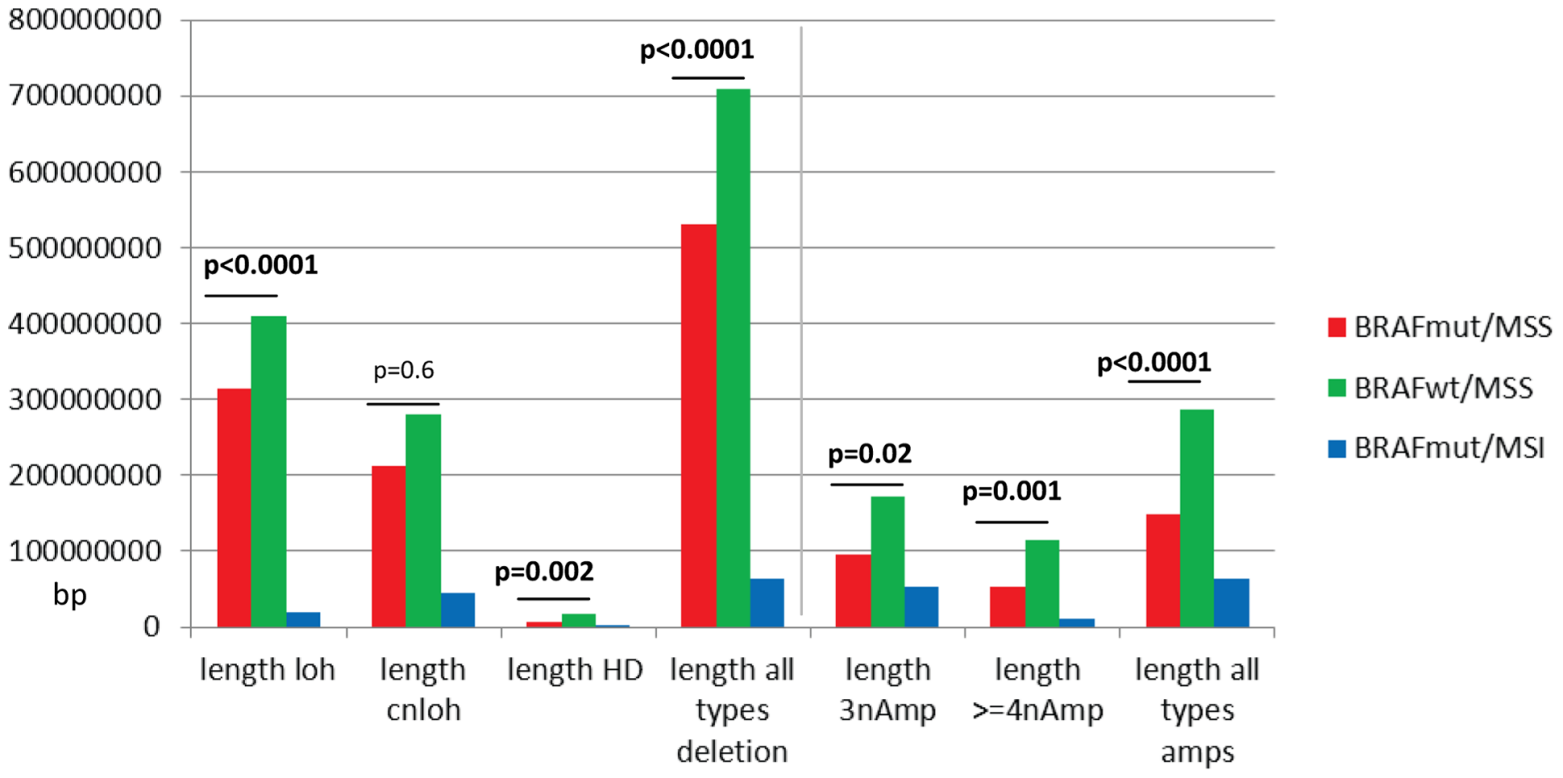
The average length of specific types of deletion and amplification copy number aberrations per cancer. There were significantly longer lengths for all events (except cnLOH) in the *BRAF*wt/MSS compared to the *BRAF*mut/MSS cohort. *BRAF*mut/MSI cancers had significantly shorter lengths for all types of events compared to MSS cancers (p<0.0001).

The average number of the specific type of either amplification or deletion CNAs per cancer demonstrated the MSS cohorts had similar rates of types of events (Table 2). However the *BRAF*wt/MSS cancers had significantly longer lengths of all types of deletion and amplification events, except cnLOH CNAs (Figure 2). *BRAF*mut/MSI had significantly lower rates and shorter lengths of all types of events compared to the MSS cohorts (Table 2, Figure 2).

### Frequency of Copy Number Aberrations According to Length

All CNAs were assessed for the fraction of coverage according to the specific chromosomal arm. Analysis of the length of all CNAs showed the vast majority were either less than 50% or longer than 95% the length of a chromosome arm for each of the three cohorts (Figure S2 in File S1). Therefore, in order to further compare frequencies of CNAs between cohorts, CNAs were considered as either whole arm (≥95% chromosomal arm length), or focal (as <50% chromosomal arm length [24], [41], [42], [43]). The remaining CNAs (50–94% chromosome arm length) were considered as regional events. Varying the threshold of the focal length from <35% to <65% chromosome arm length, still resulted in the majority of CNAs being kept in either the focal or whole length subsets, and did not alter the statistical significance of important findings (Tables S3A and S3B in File S1).

#### Whole Chromosome Arm Copy Number Aberrations

The *BRAF*wt/MSS cohort had a significantly higher propensity for whole chromosome arm CNA events at 32% compared to the *BRAF*mut/MSS cohort at 17% (p<0.0001) (Table 2). This corresponded to a significantly higher average rate of whole chromosome arm CNAs per cancer in *BRAF*wt/MSS compared to *BRAF*mut/MSS cancers (p = 0.04); and a greater average proportion of genome affected by whole arm events in BRAFwt/MSS compared to BRAFmut/MSS cancers (22% vs 12%, p = 0.02) (Table 2). The *BRAF*mut/MSI cohort had the lowest whole arm CNA rate of just 1.4 per cancer (p<0.0001); and just 3% of their genome affected by whole arm CNAs (p<0.0001) (Table 2). Within both MSS cohorts, the average number of whole arm losses were significantly greater than the average number of whole arm gains (*BRAF*mut/MSS p<0.0001, *BRAF*wt/MSS p = 0.001). *BRAF*wt/MSS cancers had significantly more whole arm gain events than BRAFmut/MSS cancers (p = 0.01) (Table 2).

#### Regional Copy Number Aberrations

Rates of regional CNAs were similar between cohorts and whilst they occurred at a lower rate compared to whole arm and focal events, their inclusion allowed for a comprehensive description of CIN across all three cohorts (Table 2). Both MSS cohorts had significantly more regional deletion than amplification events per sample (Table 2).

#### Focal Copy Number Aberrations

The *BRAF*mut/MSS cohort had the highest proportion of focal CNAs at 70.7% of all CNAs, whereas the *BRAF*wt/MSS had significantly less at 54.9% (p<0.0001). This equated to a rate of 23.2 focal CNAs per *BRAF*mut/MSS cancer, and 16.3 per *BRAF*wt/MSS cancer; the *BRAF*mut/MSI cancers had substantially fewer focal CNAs at 3.4 per cancer (p<0.0001) (Table 2). There were significantly differing average lengths of focal aberrations per cohort with 6.3 Mb for *BRAF*mut/MSS, 10.9 Mb for *BRAF*wt/MSS and 2.3 Mb for *BRAF*mut/MSI cancers (p<0.0001).

In all cohorts focal deletion CNAs, predominantly through LOH events, were significantly greater than focal amplification CNAs (*BRAF*mut/MSS p = 0.004, *BRAF*wt/MSS p = 0.03) (Table 2), (*BRAF*mut/MSI p = 0.0001). Compared to *BRAF*wt/MSS cancers, *BRAF*mut/MSS cancers showed significantly more frequent focal deletions at 18q (11/33, 33% Vs 1/18, 6%; p = 0.04) predominantly encompassing 18q21.2 which includes the *SMAD2* gene locus. Focal amplifications in *BRAF*mut/MSS cancers were also more common compared to *BRAF*wt/MSS cancers at 8q (11/33, 33% Vs 1/18, 6%; p = 0.04) predominantly at 8q24.21 covering the *Myc* locus, and 18q (7/33, 21% Vs 0/18; p = 0.04) affecting 18q11.2 (containing *GATA6* and *CTAGE).*

#### Minimal Common Regions (MCRs)

Minimal common regions were considered to include all lengths of CNAs. Both MSS cohorts showed a high rate of cancers (≥40%) with targeted deletion events at several loci previously associated with CRC, such as 18q21.1–18q21.2 (which includes *SMAD2, SMAD4*, *DCC*) and 17p13.1 (*p53*) (Table S4A in File S1). Loci not as commonly associated with CRC were also found to be deleted in a similar proportion of MSS cancers, and included 22q12.1, 22q11.1, 22q13.2, 17p12, 17p11.2, each of which contain several cancer related genes (Table S4A in File S1).

Analysis of MCRs affecting ≥20% of cancers in at least one of the two MSS cohorts revealed several loci where the rates of CNAs differed substantially between cohorts. Although after adjustment for multiple comparisons significance was no longer reached, the *BRAF*mut/MSS cancers had a high frequency of deletion CNAs compared to *BRAF*wt/MSS cancers at several loci on 17q and 6p including 17q22 (that contains cancer related genes *RNF43* and *VEZF1*), 17q24.3 (*SOX9*) and 6p25.1 (*CDYL*) (Table 3). Amplification MCRs were more common in *BRAF*mut/MSS than BRAFwt/MSS cancers at 8q24.21 (*Myc*), and 18q11.2 (*GATA6, CTAGE*) (Table 3).

**Table 3:** Minimal Common Regions (MCRs) affecting ≥20% of cancers in at least one of the *BRAF*mut/MSS or *BRAF*wt/MSS cohorts where differences in CNA frequencies were detected between MSS cohorts.

Chr Arm	Chr band start	Start position (bp)	Chr band end	End position (bp)	Length of MCR (bp)	Type of CNA	% of *BRAF *mut / MSS . n = 33	% of *BRA F*wt/ MSS. n = 18	p value	Adjusted p value	Potential Cancer Related Genes Involved
2q	q37.3	240,832,001	q37.3	242,518,000	1,686,000	Deletion	6.1	**33.3**	**0.02**	0.27	*GPC1, CAPN10, KIF1A, SEPT2, STK25*
5q	q34.3	165,279,001	q34	167,417,000	2,138,000	Deletion	27.3	55.6	0.07	0.49	*ODZ2*
6p	p25.1	4,134,083	p25.1	7,009,966	2,875,884	Deletion	**45.5**	5.6	**0.004**	0.27	*CDYL*
6p	p22.3	15,240,001	p22.3	15,916,000	676,000	Deletion	**42.4**	11.1	**0.03**	0.34	*JARID2*
6p	p21.33	31,097,001	p21.33	31,680,000	583,000	Deletion	**39.4**	5.6	**0.01**	0.27	*MICA, MICB, TNF*
6q	q16.1	99,203,001	q16.2	00,187,000	984,000	Deletion	**27.3**	5.6	0.08	0.49	*CCNC*
17q	q22	55,950,001	q22	57,384,000	1,434,000	Deletion	**57.6**	22.2	**0.02**	0.27	*RNF43, VEZF1, SEPT4, TEX14, RAD51C, PPM1E, TRIM37, SKA2*
17q	q24.1	62,839,470	q24.1	63,914,355	1,074,886	Deletion	**51.5**	22.2	0.07	0.49	*AXIN2, GNA13*
17q	q24.3	68,762,000	q24.3	70,569,000	1,807,001	Deletion	**57.6**	27.8	**0.02**	0.27	*SOX9*
17q	q25.1	70,650,001	q25.1	71,431,000	781,000	Deletion	**54.5**	22.2	**0.04**	0.38	*SDK2, SSTR2, CDC42EP4*
7p	p21.3	7,492,001	p21.1	20,814,000	13,322,000	Amp	9.1	**38.9**	**0.02**	**0.051**	*PHF14, ARF4L, ETV1, AGR2/3, BZW2, HDAC9, TWIST1, MACC1, ITGB8, ABCB5*
7q	q21.11	81,993,001	q21.11	82,532,000	539,000	Amp	3.0	**38.9**	**0.002**	**0.012**	*CACNA2D3, PCLO*
7q	q36.2	154,436,001	q36.3	159,119,000	4,683,000	Amp	3.0	**38.9**	**0.002**	**0.012**	*DPP6, INSIG1, SHH, RNF32, MNX1, LMBR1, PTPRN2, NCAPG2, VIPR2*
8q	q24.21	128,085,001	q24.21	129,127,000	1,042,000	Amp	**48.5**	16.7	**0.035**	0.079	*MYC, PVT1*
8q	q23.1	109,055,001	q23.2	109,888,000	833,000	Amp	**45.5**	16.7	0.065	0.097	*RSP02, EIF3E*
8q	q24.11	117,636,001	q24.11	118,097,000	461,000	Amp	**45.5**	16.7	0.065	0.097	*EIF3H, UTP23, RAD21*
8q	q24.22	132,704,000	q24.22	135,106,000	2,402,001	Amp	**45.5**	16.7	0.065	0.097	*EFR3A, PHF20L1, SLA, WISP1, NDRG1, ST3GAL1*
8q	q22.3	100,850,001	q22.3	102,660,000	1,810,000	Amp	**42.4**	16.7	0.07	0.097	*VPS13B, RGS22, SPAG1, RNF19A, ANKRD46, SNX31, PABPC1, YWHAZ, ZNF706, GRHL2*
13q	q14.11	40,387,001	q14.11	44,439,000	4,052,000	Amp	12.1	**66.7**	**0.0001**	0.002	*FOXO1, ELF1, DGKH, EPSTI7, ENOX1*
13q	q21.33	70,105,001	q21.33	70,950,000	845,000	Amp	21.2	**61.1**	**0.007**	0.030	*KLHL1*
18q	q11.2	19,636,000	q11.2	20,838,000	1,202,001	Amp	**21.2**	0.0	**0.04**	0.080	*GATA6, CTAGE, RBBP8, CABLES27*
20p	p11.21	24,267,001	p11.1	25,184,000	917,000	Amp	9.1	**44.4**	**0.01**	0.030	*ACSS1*
20q	q13.2	50,823,001	q13.33	56,496,000	5,673,000	Amp	18.2	**55.6**	**0.01**	0.030	*AURKA, TSHZ2, BCAS1, PFDN4, CASS4, BMP7, CTCFL, RAE1, ZBP1, CTCFL, PMEPA1*

(Benjamini-Hochberg method applied for adjusted p values).

The *BRAF*mut/MSI cancers had substantially fewer MCRs than the MSS cohorts, however they did have a comparatively high proportion of cancers (≥20%) with focal deletions at 3p14.2 (*FHIT*), 16p13.3 (*RBFOX1*) and 20p12.1 (*MACROD2)* (Table S4C in File 1).

### Different Patterns of CIN exist between the *BRAF*mut/MSS and *BRAF*wt/MSS Cancers

**Figure 3:**
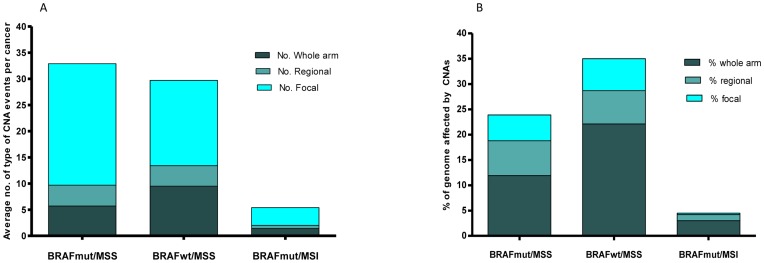
Average number of copy number aberrations (CNAs) and percentage of genome affected per MSS cohort. A) Average number of CNAs delineated by length per cancer in each MSS cohort. MSS cohorts had a similar number of overall CNAs occurring per cancer, however the *BRAF*mut/MSS cancers showed a greater number of focal CNAs, with the *BRAF*wt/MSS cancers having a greater number of whole arm events. *BRAF*mut/MSI cancers had considerably fewer CNAs of all types. B) Average percentage of genome affected by CNAs delineated by length in each MSS cohort. *BRAF*wt/MSS cancers had the greatest proportion of genome affected by CNA events, which was due to the higher number of whole arm events in this cohort. *BRAF*mut/MSS cancers showed a lower proportion of the genome affected by CNAs, which is reflective of the comparably lower rate of whole arm and higher rate of focal events that occurred compared to *BRAF*wt/MSS cancers.

Although the MSS cohorts had similar average numbers of CNAs per cancer (Figure 3A, Table 2), the *BRAF*wt/MSS cancers had the greatest proportion of genome affected by CNAs (Figure 3B). CIN in a typical *BRAF*wt/MSS cancer predominantly occurred via whole chromosome arm events, whereas CIN in *BRAF*mut/MSS cancers largely correlated with frequent focal CNAs which resulted in a smaller proportion of genome affected (Figures 3A and 3B).

**Figure 4:**
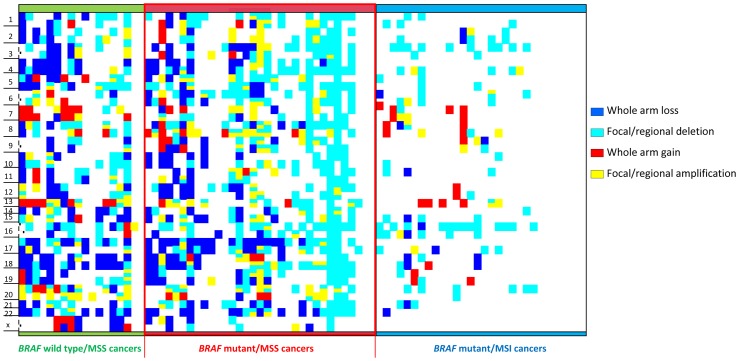
Heat map showing the distribution of whole chromosome arm and focal copy number aberrations across the cohorts. Sample heterogeneity occurred within cohorts however a focal pattern is evident in the *BRAF*mut/MSS and a whole arm pattern is present in the *BRAF*wt/MSS cohort.

Figure 4 shows the genome wide distribution of CNAs across the three cohorts according to the type and length of event that occurred at a particular chromosome arm. Although the MSS cohorts do show sample heterogeneity, the predominantly focal pattern of CIN is evident in *BRAF*mut/MSS cancers, and this contrasts to the whole chromosome arm pattern seen in *BRAF*wt/MSS cancers.

## Discussion

This study has shown that *BRAF*mut/MSS colorectal cancers predominantly harbour focal or targeted CNAs, whereas the *BRAF*wt/MSS colorectal cancers have significantly more frequent whole chromosome arm CNAs. This results in a greater average percentage of genome affected by CIN in *BRAF*wt/MSS compared to *BRAF*mut/MSS cancers. *BRAF*wt/MSS cancers show a similarly high percentage of the genome affected by whole arm CNAs as a previous report of a large series of different cancer types, including CRC [41]. Comparatively, the *BRAF*mut/MSS cohort has a significantly smaller proportion of their CIN affected genomes covered by whole arm events. Overall these observations identify that *BRAF*mut/MSS cancers represent a more ‘focal pattern’ of CIN, whereas *BRAF*wt/MSS cancers display a ‘whole chromosome arm’ pattern of CIN.

Across all cohorts, the frequency of deletion CNAs exceeded amplification events for all types of CNAs. This difference may reflect a greater selection for deletions which could be tumour promoting and involve more simple mechanisms of acquisition, whereas amplifications may require more complex interactions with homologous and non-homologous chromosomes [44].

Whole chromosome arm CNAs were significantly more common in *BRAF*wt/MSS than in *BRAF*mut/MSS cancers. Whole chromosome arm CNAs can promote tumourigenesis through the gain of oncogenes and loss of tumour suppressors on a large scale [19]. However, whole chromosome arm CIN is also linked with cancer repression where the reverse of cancer promoting effects occur, and there is an overabundant loss of oncogenic factors and gain of tumour suppressive effects [19]. Additionally, increased chromosome copy number can lead to excessive protein production which may place greater metabolic stress on the cancer cell and ultimately reduce their rate of cellular growth and proliferation [19], [45], [46], [47]. Whether the propensity of whole arm CNAs may be contributing to the less adverse nature of BRAFwt/MSS cancers compared to BRAFmut/MSS cancers through mechanisms described above, may warrant further investigation.

The aggressive *BRAF*mut/MSS cancers had a significantly higher rate of focal CNAs across their genomes. There have been previous reports of early compared to late stage cancers harbouring more whole arm compared to focal CNAs [48], where stage I breast cancers were found to have more frequent whole chromosome arm CNAs compared to stage II/III breast cancers which had smaller, more complex events [49]. Furthermore, a detrimental clinical outcome in melanoma has been associated with a greater frequency of focal CNAS compared to whole chromosome arm events [50]. Potentially these complex, sub-chromosomal events may be facilitating cancer progression by specifically targeting key drivers of tumourigenesis.

Different mechanisms relating to the origin of either whole chromosome or focal CNAs exist. It has been commonly reported that CIN involving whole chromosomes is due to errors relating to chromosome segregation during mitosis [19], [51]. These errors are more likely to be those involving dysregulation of kinetochore-microtubule attachments, termed merotely, where a chromosome attached to both spindle poles mis-segregates at anaphase and results in whole chromosome aneuploidy [20]. The ‘focal’ pattern of CIN we have identified in *BRAF*mut/MSS cancers may associate with ‘structural’ CIN which involves structural sub-chromosomal rearrangements including deletions, amplifications and translocations [20]. The causes of these types of structural aberrations may involve dysfunctional repair processes of double strand breaks by homologous recombination and the error prone non-homologous end joining [52]. Potentially many of the particularly complex patterns of structural aberrations may not be driver mechanisms in tumourigenesis but instead could be consequences of these disrupted DNA damage and repair processes. Studies of further genetic abnormalities unique to these specific CRC subgroups that could predispose to their respective patterns of CIN may be warranted.

We have previously found that the CpG Island Methylator Phenotype (CIMP) and CIN can co-exist in *BRAF*mut/MSS cancers [22]. Potentially, the degree of methylation present and the subsequent effects on the extent of chromatin compaction may relate to the different rates of focal and whole arm CNAs observed between *BRAF*mut/MSS and *BRAF*wt/MSS cancers. Regional hypermethylation as present in CIMP positive cancers associates with increased levels of condensed chromatin, whereas widespread hypomethylation is present in cancers with a more open chromatin conformation [53]. This and our previous studies have found a substantial rate of *BRAF*mut/MSS cancers to be CIMP high [22], [36], which may confer a more closed chromatin structure in these cancers. Global hypomethylation is well documented in CRC where it can associate with CIN and affects predominantly *BRAF* wild type cancers [54]. A study found in regions with predominantly open or relaxed chromatin, repair mechanisms following double strand breaks were quicker to act due to greater accessibility of repair enzymes to the damaged site and subsequently resulted in less chromosome fragmentation [55]. These findings could help to account for the reduced rate of focal CNAs found in the *BRAF*wt/MSS cohort. The majority of *BRAF*mut/MSS cancers were CIMP high and demonstrated a ‘focal pattern’ of CIN, which may suggest that a condensed chromatin structure contributes to a propensity of focal CNAs.

CIN was evident in the majority of *BRAF*mut/MSI cancers, but affected a much smaller proportion of the genome compared to MSS cancers. Several genomic regions containing fragile sites, such as the *FHIT* gene locus at 3p14.2, *RBFOX1* at 16p13.3 and *MACROD2* at 20p12.1 that were targeted for deletion in MSS cancers, were also relatively commonly deleted in *BRAF*mut/MSI cancers [56], [57]. The lower degree of CIN present in MSI cancers may relate to findings that the onset of MSI is an early event in the development of MSI/CIMP positive cancers [58], and as this type of genomic instability is already present, there may be redundancy for the development of further genomic instability through CIN.

As well as distinct variations in the pattern of CIN displayed between the two MSS cohorts, analysis of the minimal common regions (MCRs) of CIN revealed differential rates of either deletion or amplification CNAs occurring at certain genomic loci between them. Many of those more frequent in *BRAF*mut/MSS cancers, for example deletions at 6p25.1-6p21.33 and at specific loci on 17q where several Wnt regulatory genes reside (*RNF43*, *AXIN2* and *SOX9)* [59], [60], [61], have not commonly been associated with CRC. Additionally, *BRAF*mut/MSS cancers had a higher frequency of targeted amplification of 8q24.13 that contains the Wnt signalling effector, *Myc.* These Wnt pathway related genes that may be specifically targeted in *BRAF*mut/MSS cancers could be an alternative mechanism in promotion of the Wnt signal in this cancer subtype. Amplification CNAs occurred at 18q11.2 in *BRAF*mut/MSS cancers, whereas non-specific whole arm deletion events affected this region in *BRAF*wt/MSS cancers. The 18q11.2 locus harbours two genes, *GATA6* and *CTAGE* that have previously been reported to be amplified and upregulated in gastrointestinal cancers [39], [43] including metastatic CRC [62]. Extended studies of these loci where greater rates of MCRs occur in *BRAF*mut/MSS compared to *BRAF*wt/MSS cancers may be indicated to ascertain whether these are driver mechanisms that may uniquely promote tumourigenesis in the *BRAF*mut/MSS cohort.

This study has determined that a substantial presence of genome-wide CIN exists in the aggressive *BRAF*mut/MSS cancers of the serrated neoplastic pathway. Significantly different patterns of CIN were found between the two MSS cohorts. *BRAF*mut/MSS cancers were found to harbour frequent focal length CNAs and therefore display a ‘focal pattern’ of CIN suggestive of commonly occurring structural rearrangements. Alternatively, the greater presence of whole arm CNAs in the *BRAF*wt/MSS cancers indicate they have a ‘whole chromosome arm pattern’ of CIN that may be due to dysfunctional mitotic events. Overall these findings suggest that either presence or absence of the *BRAF* V600E mutation could potentially affect subsequent acquisition of genomic instability in these subgroups of CRC. Extended studies to ascertain the clinical impact of the different patterns of CIN identified in these cancer subgroups may be warranted. Additionally, specific loci not as commonly associated with CRC that were more frequently affected by CIN in *BRAF*mut/MSS cancers were found, and this could help in the identification of molecular events that may correlate with the aggressive nature of these *BRAF*mut/MSS colorectal cancers.

## Supporting Information

File S1 **Contains the files:** Figure S1. Automatic inclusion of cancer samples with tumour percentage ≥40% as estimated by SiDCoN. Figure S2. Frequency of copy number aberrations occurring delineated by their fraction of chromosome arm per cohort. Table S1. Verification of the inclusion of cancers that had a tumour percentage ≤40% in this study by analysing the presence of cancer related molecular changes. Table S2. Data and statistical analysis with the exclusion of cancers with <40% tumour content. Table S3A. Data and statistical analysis of cohorts when threshold of focal CNA group is changed to <35% chromosome arm length. Table S3B. Data and statistical analysis of cohorts when threshold of focal CNA group is changed to <65% chromosome arm length. Table S4. Minimal Common Regions (MCRs) of copy number aberrations affecting ≥20% of cancers in at least one of the *BRAF*mut/MSS or *BRAF*wt/MSS cohorts.
(DOCX)
