# Supplementary material for: Microsatellite Stable Colorectal Cancers Stratified by the BRAF V600E Mutation Show Distinct Patterns of Chromosomal Instability
Source: PLoS One. 2014 Mar 20;9(3):e91739. doi: 10.1371/journal.pone.0091739 (PMC3961279; doi:10.1371/journal.pone.0091739)
Supplement: File S1 — Contains the files: Figure S1. Automatic inclusion of cancer samples with tumour percentage ≥40% as estimated by SiDCoN. Figure S2. Frequency of copy number aberrations occurring delineated by their fraction of chromosome arm per cohort. Table S1. Verification of the inclusion of cancers that had a tumour percentage ≤40% in this study by analysing the presence of cancer related molecular changes. Table S2. Data and statistical analysis with the exclusion of cancers with <40% tumour content. Table S3A. Data and statistical analysis of cohorts when threshold of focal CNA group is changed to <35% chromosome arm length. Table S3B. Data and statistical analysis of cohorts when threshold of focal CNA group is changed to <65% chromosome arm length. Table S4. Minimal Common Regions (MCRs) of copy number aberrations affecting ≥20% of cancers in at least one of the BRAFmut/MSS or BRAFwt/MSS cohorts. (DOCX) [file pone.0091739.s001.docx]

**Supporting Information**

Figure S1: Automatic inclusion of cancer samples with tumour percentage ≥40% as estimated by SiDCoN ([1](#_ENREF_1)).
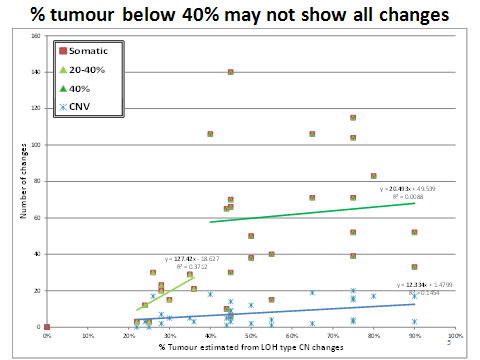


% Tumour estimates from LOH type CN changes

^140^

^120^

^100^

^80^

^20^

^40^

^60^

Number of changes

^0^

^0 10 20 30 40 50 60 70 80 90^

Y=20.5x+49

**R^2^=0.009**

Y=12.3x+1.5

R^2^=0.1

Y=127.4x+18.6

R^2^=0.4

This shows that cancers below 40% tumour content as estimated from LOH CNAs have a reduced number of CNAs compared to those above 40%. Therefore, presence of other tumour related factors were ascertained in order to verify inclusion of the <40% samples. Statistics relating to important findings were similar with exclusion of these cancers compared to when they were included (Supplementary data, Table 1).

Figure S2: The frequency of copy number aberrations occurring delineated by their fraction of chromosome arm per cohort.

Table S1: Verification of the inclusion of cancers that had a tumour percentage ≤40% ([1](#_ENREF_1)) in this study by analysing the presence of cancer related molecular changes ([2](#_ENREF_2)).

| **Cohort** | **SNP Array Sample No.** | **% tumour content** | **No. of CNAs** | **MSI Status** | ***BRAF* V600E Status** | ***p53* Status** | ***KRAS* Status** | ***MGMT* Meth PMR** | **CIMP *MINT1* PMR** | **CIMP *MINT2* PMR** | **CIMP *MINT31* PMR** | **CIMP *MLH1* PMR** | **CIMP *P16* PMR** | **CIMP *NEUROG1* PMR** | **CIMP *SOCS1* PMR** | **CIMP *CACNAIG* PMR** | **CIMP *IGF2* PMR** | **CIMP *RUNX*3 PMR** | **LOH Status** |
| --- | --- | --- | --- | --- | --- | --- | --- | --- | --- | --- | --- | --- | --- | --- | --- | --- | --- | --- | --- |
| ***BRAF* mut/ MSI** | 8 | NA | 0 | **MSI** | mut | wt | wt | 0 | **19** | **21** | 9 | **31** | **23** | 8 | 3 | 0 | 7 | **18** | - |
|  | 38 | NA | 0 | **MSI** | mut | wt | wt | 0 | **26** | **24** | **14** | **26** | **26** | **15** | **21** | **10** | **14** | **23** | - |
|  | 42 | NA | 0 | **MSI** | mut | wt | wt | **23** | **30** | **29** | **29** | **30** | 0 | **57** | 1 | **39** | **59** | **59** | - |
|  | 44 | NA | 0 | **MSI** | mut | wt | wt | 0 | 7 | 7 | 6 | 8 | **13** | 9 | 0 | 3 | 6 | 7 | 0 |
|  | 46 | NA | 0 | **MSI** | mut | **Silent mut R231R** | wt | 9 | **14** | **20** | **17** | 9 | 7 | 9 | 0 | **17** | **16** | 9 | - |
|  | 87 | NA | 0 | **MSI** | mut | wt | wt | 0 | 8 | **13** | 6 | 8 | 0 | 8 | 0 | **17** | 0 | 3 | 0 |
|  | 156 | NA | 0 | **MSI** | mut | wt | wt | 0 | 6 | **11** | 9 | 8 | **14** | 3 | 8 | 3 | 6 | 8 | 0 |
|  | 26 | 35 | 1 | **MSI** | mut | **mut R248Q** | wt | **37** | **43** | **35** | **41** | **32** | **34** | **30** | **31** | **27** | **42** | **33** | **17p** |
|  | 48 | 20 | 1 | **MSI** | mut | wt | wt | 0 | **11** | **16** | 9 | 8 | 0 | 8 | 1 | 5 | **13** | 7 | 0 |
|  | 85 | 30 | 4 | **MSI** | mut | wt | wt | **14** | 9 | **22** | **19** | **28** | 6 | 0 | 7 | **11** | **17** | **28** | - |
|  | 111 | 30 | 6 | **MSI** | mut | wt | wt | **28** | **21** | **21** | **27** | **23** | **16** | **18** | 9 | **22** | **23** | **25** | 0 |
|  | 166 | 28 | 6 | **MSI** | mut | wt | wt | 0 | **39** | **39** | **31** | **32** | **29** | **34** | **28** | **31** | **49** | **43** | **18q** |
| ***BRAF* mut/ MSS** | 58 | 20 | 1 | MSI-L | mut | wt | wt | **14** | **26** | **19** | **11** | 0 | 0 | 9 | 1 | 2 | 7 | **12** | 0 |
|  | 126 | 20 | 0 | MSS | mut | wt | wt | 0 | **48** | **11** | 3 | 0 | 0 | 1 | 0 | 0 | 1 | 0 | 0 |
|  | 138 | NA | 0 | MSI-L | mut | wt | wt | 0 | **19** | **18** | 5 | **13** | **14** | 5 | 9 | **11** | **11** | 0 | 0 |
|  | 14 | 30 | **15** | MSS | mut | wt | wt | 0 | **10** | **26** | 1 | 0 | 0 | **10** | 0 | 0 | 1 | 0 | 0 |
|  | 52 | 35 | **19** | MSS | mut | NA | wt | **-** | **-** | **-** | - | **-** | **-** | - | - | **-** | **-** | - | **18q 8p** |
|  | 54 | 30 | **13** | MSI-L | mut | **mut R273H** | wt | 0 | **11** | **18** | **17** | 3 | **17** | **14** | 0 | **15** | **27** | **10** | **8p** |
|  | 70 | 24 | **6** | MSS | mut | **mut V173M** | wt | 0 | 7 | **13** | 5 | 0 | 7 | 7 | 0 | 0 | 0 | 0 | 0 |
|  | 82 | 25 | **19** | MSI-L | mut | **mut R248W** | wt | 0 | 1 | **27** | **11** | 0 | 0 | **27** | 0 | 2 | 0 | 7 | **17p 5q** |
|  | 100 | 30 | **10** | MSS | mut | **silent mut E171E** | wt | 0 | **14** | **25** | **20** | 0 | **10** | **11** | 2 | **21** | **23** | **17** | 0 |
|  | 130 | 30 | **18** | MSI-L | mut | **mut R282W** | wt | 0 | 0 | **11** | 5 | 0 | 0 | 0 | 0 | 0 | 0 | 0 | **5q** |
|  | 150 | 30 | **3** | MSI-L | mut | wt | wt | 3 | **34** | **47** | **67** | **16** | **47** | **52** | **18** | **36** | **45** | **52** | **8p** |
| ***BRAF*wt/MSS** | 148 | NA | 0 | MSS | wt | **silent mut R213R** | wt | 0 | 1 | 0 | 0 | 0 | 0 | 0 | 0 | 0 | 0 | 0 | 0 |
|  | 158 | 36 | 1 | MSI-L | wt | wt | wt | 0 | 0 | 3 | 0 | 0 | 0 | 6 | 0 | 0 | 0 | 0 | **18q 5q** |

Bold print indicates the presence of validating molecular changes ([3](#_ENREF_3), [4](#_ENREF_4)) which verifies these cancers had adequate tumour content enabling them to be included in this study.

Table S2: Data and statistical analysis with the exclusion of cancers with <40% tumour content.

|  | ***BRAF*mut/MSS** | P value within BRAF mut/ MSS | ***BRAF*wt/MSS** | P value within BRAFwt/ MSS | **P Value between MSS Cohorts** | ***BRAF*mut/MSI** | **P Value between all 3 cohorts** |
| --- | --- | --- | --- | --- | --- | --- | --- |
| **n** | **22** | **-** | **16** | **-** | **-** | **18** | - |
| **Total Number of CNAs in Cohort** | 980 | - | 535 | - | - | 144 | - |
| **Average Number CNAs per Cancer** | 44.5 | - | 33.4 | - | 0.21 | 8 | **<0.0001** |
| **Average Length of CNA (Mb)** | 19.4Mb | - | 33.4Mb | - | **0.002** | 22.7Mb | **0.0037** |
| **Median Fraction of CNA over chromosome arm lengths** | 0.06 | - | 0.39 | - | **<0.0001** | 0.02 | **<0.0001** |
| **Average Fraction of CNA over chromosome arm lengths** | 0.3 | - | 0.5 | - | **<0.0001** | 0.36 | **<0.0001** |
| **Deletion CNAs in Cohort** | 729/980=74.4% | - | 372/535=69.5% | - | **<0.05** | 107/144=74.3% | 0.12 |
| **Amplification CNAs in Cohort** | 251/980=25.6% | - | 163/535=30.5% | - | - | 37/144=25.7% | **-** |
| **Average No. Deletion CNAs per cancer** | 33.1 | **<0.0001** | 23.3 | **0.0006** | 0.14 | 5.9 | **<0.0001** |
| **Average No. Amplification CNAs per cancer** | 11.4 |  | 10.2 |  | 0.89 | 2.1 | **0.002** |
| **Av % of Genome Affected by CNAs per cancer** | 30.3% | - | 39.2% | - | **<0.0001** | 9.8% | **<0.0001** |
| **Av % of Genome Affected by Deletion CNAs per cancer** | 23.2% | **<0.0001** | 25.5.0% | 0.013 | 0.87 | 3.3% | **<0.0001** |
| **Av % of Genome Affected by Amplification CNAs per cancer** | 7.06% |  | 13.7% |  | 0.087 | 3.1% | **0.006** |
| **Whole Chromosome Arm CNAs in Cohort** | 150/980=15.3% | - | 170/535=31.8% | - | **<0.0001** | 38/144=26.4% | **<0.0001** |
| **Regional CNAs in Cohort** | 112/980=11.4% | - | 71/535=13.3% | - | - | 13/144=9.0% | 0.32 |
| **Focal CNAs in Cohort** | 718/980=73.2% | - | 294/535=55.0% | - | **<0.0001** | 93/144=64.6% | **<0.0001** |
| **Average No. Whole Arm CNAs per cancer** | 6.8 | **<0.0001** | 10.6 | **0.0009** | 0.11 | 2.1 | **0.0003** |
| **Average No. Regional CNAs per cancer** | 5.1 |  | 4.4 |  | 0.77 | 0.7 | **<0.0001** |
| **Average No. Focal CNAs per cancer** | 32.6 |  | 18.4 |  | **0.038** | 5.2 | **<0.0001** |
| **Av. % Genome Affected by Whole Arm CNAs** | 14.7% | 0.40 | 24.8% | **0.0005** | 0.075 | 4.6% | **0.0004** |
| **Av. % Genome Affected by Regional CNAs** | 8.9% |  | 7.4% |  | 0.71 | 1.3% | **0.0003** |
| **Av. % Genome Affected by Focal CNAs** | 6.7% |  | 7.0% |  | 0.98 | 0.4% | **0.0002** |

Table S3A: Data and statistical analysis of cohorts when threshold of focal CNA group is changed to <35% chromosome arm length.

|  | ***BRAF*mut/MSS** | P value within BRAF mut/ MSS | ***BRAF*wt/MSS** | P value within BRAFwt/ MSS | **P Value between MSS Cohorts** | ***BRAF*mut/MSI** | **P Value between all 3 cohorts** |
| --- | --- | --- | --- | --- | --- | --- | --- |
| **n** | 33 | - | 18 | - | - | 30 | - |
| **Whole Chromosome Arm CNAs in Cohort** | 187/1084 (17.3%) | - | 171/536 (31.9%) | - | **<0.0001** | 41/162 (25.3%) | **<0.0001** |
| **Regional CNAs in Cohort** | 183/1084 (16.9%) | - | 108/536 (20.1%) | - | - | 20/162 (12.3%) | 0.053 |
| **Focal CNAs in Cohort** | 714/1084 (65.9%) | - | 257/536 (47.9%) | - | **<0.0001** | 101/162 (62.3%) | **<0.0001** |
| **Average No. Whole Arm CNAs per cancer** | 5.7 | **<0.0001** | 9.5 | **0.03** | **0.04** | 1.4 | **<0.0001** |
| **Average No. Regional CNAs per cancer** | 5.5 |  | 6.0 |  | 0.92 | 0.7 | **<0.0001** |
| **Average No. Focal CNAs per cancer** | 21.6 |  | 14.3 |  | 0.27 | 3.4 | **0.0001** |
| **Av. % Genome Affected by Whole Arm CNAs** | 11.9% | **0.0005** | 22.1% | **<0.0001** | **0.018** | 3.0% | **<0.0001** |
| **Av. % Genome Affected by Regional CNAs** | 8.4% |  | 8.8% |  | 0.99 | 1.2% | **<0.0001** |
| **Av. % Genome Affected by Focal CNAs** | 3.5% |  | 4.0% |  | 0.83 | 0.2% | **<0.0001** |

Table S3B**:** Data and statistical analysis of cohorts when threshold of focal CNA group is changed to <65% chromosome arm length.

|  | ***BRAF*mut/MSS** | P value within BRAF mut/ MSS | ***BRAF*wt/MSS** | P value within BRAFwt/ MSS | **P Value between MSS Cohorts** | ***BRAF*mut/MSI** | **P Value between all 3 cohorts** |
| --- | --- | --- | --- | --- | --- | --- | --- |
| **n** | 33 | - | 18 | - | - | 30 | - |
| **Whole Chromosome Arm CNAs in Cohort** | 187/1084 (17.3%) | - | 171/536 (31.9%) | - | **<0.0001** | 41/162 (25.3%) | **<0.0001** |
| **Regional CNAs in Cohort** | 82/1084 (7.6%) | - | 48/536 (9.0%) | - | - | 14/162 (8.6%) | 0.6 |
| **Focal CNAs in Cohort** | 815/1084 (75.2%) | - | 317/536 (59.1%) | - | **<0.0001** | 107/162 (66.1%) | **<0.0001** |
| **Average No. Whole Arm CNAs per cancer** | 5.7 | **<0.0001** | 9.5 | **<0.0001** | **0.04** | 1.4 | **<0.0001** |
| **Average No. Regional CNAs per cancer** | 2.4 |  | 2.7 |  | 0.95 | 0.5 | **0.0002** |
| **Average No. Focal CNAs per cancer** | 24.7 |  | 17.6 |  | 0.35 | 3.6 | **0.0001** |
| **Av. % Genome Affected by Whole Arm CNAs** | 11.9% | **0.04** | 22.1% | **<0.0001** | **0.018** | 3.0% | **<0.0001** |
| **Av. % Genome Affected by Regional CNAs** | 4.9% |  | 5.1% |  | 0.99 | 1.0% | **0.002** |
| **Av. % Genome Affected by Focal CNAs** | 7.1% |  | 7.7% |  | 0.90 | 0.4% | **<0.0001** |

Varying the threshold of the focal groups length from <35% to <65% did not alter the distribution of CNAs into mainly the focal or whole arm group across the 3 cohorts, or did not alter the statistical significance of CNA occurrence between cohorts.

Table S4: Minimal Common Regions (MCRs) of copy number aberrations affecting ≥20% of cancers in at least one of the *BRAF*mut/MSS or *BRAF*wt/MSS cohorts (Benjamini-Hochberg method applied for adjusted p values).

1. Deletion MCRs within MSS cohorts

| **Chr Arm** | **Chr band start** | **Start position (bp)** | **Chr band end** | **End position (bp)** | **Length MCR (bp)** | **Type of CNA** | **% of *BRAF*mut/ MSS cancers affected**  **(n=33)** | **% of *BRAF*wt/ MSS cancers affected (n=18)** | **p Value** | **Adjusted p value** | **Potential Cancer Related Genes Involved** |
| --- | --- | --- | --- | --- | --- | --- | --- | --- | --- | --- | --- |
| 1p | 1p36.13 | 16,492,001 | 1p36.13 | 17,521,000 | 1,029,000 | Deletion | 27.3 | 50.0 | 0.13 | 0.63 | *NBPF1, CROCC, SDHB, MFAP2, PADI2* |
| 1p | p21.1 | 101,914,838 | p21.1 | 102,742,000 | 827,163 | Deletion | 30.3 | 38.9 | 0.55 | 0.90 | *OLFM3* |
| 1q | q31.2 | 192,390,001 | q31.2 | 192,883,000 | 493,000 | Deletion | 12.1 | 27.8 | 0.25 | 0.84 | *RGS2* |
| 2p | p24.2 | 18,580,001 | p21 | 47,267,000 | 28,687,000 | Deletion | 9.1 | 27.8 | 0.11 | 0.61 | *MATN3, APOB, PUM2, WDR35, SDC1, RHOB, GDF7, ATAD2, ALK, NLRC4, BIRC6, LTBP1, CRIM1, PRKD3, CDKL4, EML4, ABCG5/8, LRPPRC* |
| 2q | q37.3 | 240,832,001 | q37.3 | 242,518,000 | 1,686,000 | Deletion | 6.1 | **33.3** | **0.02** | 0.27 | *GPC1, CAPN10, KIF1A, SEPT2, STK25* |
| 3p | p14.2 | 60,037,001 | p14.2 | 61,178,000 | 1,141,000 | Deletion | 36.4 | 27.8 | 0.20 | 0.79 | *FHIT* |
| 3q | q22.3 | 135,989,001 | q22.3 | 136,700,000 | 711,000 | Deletion | 18.2 | 22.2 | 0.73 | 0.94 | *PCCB, STAG1, TMEM22* |
| 3q | q25.3 | 155,370,001 | q25.4 | 157,187,000 | 1,817,000 | Deletion | 24.2 | 22.2 | 1.0 | 1.00 | *GMPS, TIPARP* |
| 4p | p16.3 | 2,839,001 | p16.3 | 3,804,000 | 965,000 | Deletion | 33.3 | 44.4 | 0.55 | 0.90 | *GRK4, RGS12, DOK7* |
| 4p | p16.1 | 11,133,001 | p15.33 | 11,624,000 | 491,000 | Deletion | 30.3 | 38.9 | 0.55 | 0.90 | *HS3STI* |
| 4p | p15.1 | 29,304,000 | p15.1 | 29,682,000 | 378,001 | Deletion | 30.3 | 38.9 | 0.55 | 0.90 | *-* |
| 4p | p14 | 37,629,001 | p14 | 39,082,000 | 1,453,000 | Deletion | 30.3 | 38.9 | 0.55 | 0.90 | *TBC1D1, KLF3, TLR10* |
| 4p | p12 | 45,700,001 | p12 | 47,244,000 | 1,544,000 | Deletion | 30.3 | 38.9 | 0.55 | 0.90 | *-* |
| 4q | q12 | 53,000,001 | q12 | 53,457,000 | 457,000 | Deletion | 36.4 | 44.4 | 0.76 | 0.94 | *-* |
| 4q | q13.1 | 65,765,001 | q13.1 | 66,569,000 | 804,000 | Deletion | 39.4 | 44.4 | 0.54 | 0.90 | *EPHA5* |
| 4q | q13.3 | 72,304,001 | q13.1 | 72,700,000 | 396,000 | Deletion | 36.4 | 44.4 | 0.76 | 0.94 | *SLC4A4* |
| 4q | q22.1 | 91,210,000 | q22.1 | 92,140,000 | 930,001 | Deletion | 33.3 | 44.4 | 0.55 | 0.90 | *FAM190A* |
| 4q | q26.33 | 117,000,000 | q27 | 121,686,000 | 4,686,001 | Deletion | 36.4 | 44.4 | 0.76 | 0.94 | *FABP2, MAD2LI* |
| 4q | q34.3 | 180,000,000 | q35.1 | 183,907,000 | 3,907,001 | Deletion | *36.4* | *50.0* | 0.39 | 0.90 | *ODZ3* |
| 5p | p13.3 | 30,888,001 | p13.3 | 31,800,000 | 912,000 | Deletion | 24.2 | 16.7 | 0.73 | 0.94 | *CDH6, DROSHA* |
| 5q | q11.2 | 58,159,000 | q11.2 | 58,635,000 | 476,001 | Deletion | 39.4 | 38.9 | 1.0 | 1.00 | *PDE4D* |
| 5q | q34.3 | 165,279,001 | q34 | 167,417,000 | 2,138,000 | Deletion | 27.3 | 55.6 | 0.07 | 0.49 | *ODZ2* |
| 5q | q35.3 | 176,628,001 | q35.3 | 178,153,000 | 1,525,000 | Deletion | 30.3 | 50.0 | 0.23 | 0.84 | *NSD1, MXD3, GRK6, DOK3, COL23A1* |
| 6p | p25.1 | 4,134,083 | p25.1 | 7,009,966 | 2,875,884 | Deletion | **45.5** | 5.6 | **0.004** | 0.27 | *CDYL* |
| 6p | p22.3 | 15,240,001 | p22.3 | 15,916,000 | 676,000 | Deletion | **42.4** | 11.1 | **0.03** | 0.34 | *JARID2* |
| 6p | p21.33 | 31,097,001 | p21.33 | 31,680,000 | 583,000 | Deletion | **39.4** | 5.6 | **0.01** | 0.27 | *MICA, MICB, TNF* |
| 6q | q16.1 | 99,203,001 | q16.2 | 00,187,000 | 984,000 | Deletion | **27.3** | 5.6 | **0.08** | 0.49 | *CCNC* |
| 8p | p23.3 | 177,001 | p23.2 | 3,896,000 | 3,719,000 | Deletion | 33.3 | 44.4 | 0.55 | 0.90 | *ERICH1, DLGAP2, ARHGEF10* |
| 8p | p21.3 | 19,997,001 | p21.3 | 22,968,000 | 2,971,000 | Deletion | 39.4 | 38.9 | 1.0 | 1.00 | *BMP1, GFRA2, DOK2, NPM2, FGF17, PIWIL2, SLC39A14, SORBS3, PDLIM2, EGR3, RhoBTB2, TNFRSF10B* |
| 8p | p12 | 31,759,001 | p12 | 32,526,000 | 767,000 | Deletion | 36.4 | 33.3 | 1.0 | 1.00 | *NRG1* |
| 8p | p12 | 34,866,001 | p11.23 | 36,553,000 | 1,687,000 | Deletion | 33.3 | 33.3 | 1.0 | 1.00 | *UNC5D* |
| 9p | p21.1 | 29,174,001 | p21.1 | 29,544,000 | 370,000 | Deletion | 27.3 | 22.2 | 0.75 | 0.94 | *-* |
| 10p | p12.1 | 29,290,001 | p11.23 | 29,628,000 | 338,000 | Deletion | 27.3 | 22.2 | 0.75 | 0.94 | *-* |
| 10q | q23.31 | 89,310,001 | q23.31 | 90,030,000 | 720,000 | Deletion | 33.3 | 27.8 | 0.76 | 0.94 | *MINPP1, PAPSS2, KLLN, PTEN* |
| 10q | q23.33 | 94,966,001 | q24.1 | 95,959,000 | 993,000 | Deletion | 33.3 | 16.7 | 0.33 | 0.90 | *MYOF, CEP55, RBP4, PLCE1* |
| 11q | q12.3 | 61,746,001 | q13.2 | 66,061,000 | 4,315,000 | Deletion | 18.2 | 22.2 | 0.73 | 0.94 | *INCENP, ASRGL1, MTA2, GANAB, UBXN1, STX5, LGALS12, RTN3, STIP1, FERMT3, VEGFB, ESRRA, MAN1, EHD1, SNX15, CDCA5, TM7SF2, FRMD8, NEAT1, SCYL1, LTBP3, MAP3K11, AIPA1, RELA, KATS, OVOL1, CFL1, BANF1,FIBP, EFEMP2, SF3B2, PACS1* |
| 11q | q22.1 | 100,413,001 | q22.1 | 101,160,000 | 747,000 | Deletion | 18.2 | 27.8 | 0.49 | 0.90 | *PGR* |
| 12p | p13.1 | 12,829,001 | p13.1 | 14,318,000 | 1,489,000 | Deletion | 30.3 | 16.7 | 0.34 | 0.90 | *CDKN1B, DDX47, RMP1, GRIN2B* |
| 12p | p12.3 | 18,135,001 | p12.3 | 18,305,000 | 170,000 | Deletion | 30.3 | 16.7 | 0.34 | 0.90 | *-* |
| 12p | p11.22 | 27,844,001 | p11.22 | 28,139,000 | 295,000 | Deletion | 33.3 | 22.2 | 0.53 | 0.90 | *PPFIBP1, PTHLH* |
| 14q | q32.12 | 94,827,001 | q32.3 | 96,897,000 | 2,070,000 | Deletion | 27.3 | 38.9 | 0.53 | 0.90 | *SERPINA1, DICER1, CLMN, TCL6, BDKRB2, AK7* |
| 15q | q11.23 | 23,684,000 | q11.2 | 25,107,000 | 1,423,001 | Deletion | 33.3 | 33.3 | 1.0 | 1.00 | *MKRN3, SNRPN* |
| 15q | q14 | 36,595,000 | q14 | 37,400,000 | 805,001 | Deletion | 33.3 | 33.3 | 1.0 | 1.00 | *MEIS2* |
| 16p | p13.3 | 5,831,001 | p13.3 | 7,534,000 | 1,703,000 | Deletion | 45.5 | 33.3 | 0.55 | 0.90 | *RBFOX1* |
| 16q | q23.1 | 78,106,000 | q23.2 | 79,335,000 | 1,229,001 | Deletion | 21.2 | 11.1 | 0.46 | 0.90 | *WWOX* |
| 17p | p13.1 | 5,658,000 | p13.1 | 7,722,000 | 2,064,001 | Deletion | 57.6 | 72.2 | 0.37 | 0.90 | *p53, XAF1, BCL6B, DLG4, ACADVL, EIF5A, YBX2, TNK1, FGF11, TNFS12/3, SHBG, WRAP53* |
| 17p | p12 | 11,216,001 | p12 | 12,436,000 | 1,220,000 | Deletion | 69.7 | 72.2 | 1.0 | 1.00 | *DNAH9, ZNF18, MAP2K4* |
| 17p | p11.2 | 16,342,001 | p11.2 | 17,708,000 | 1,366,000 | Deletion | 69.7 | 66.7 | 1.0 | 1.00 | *TNFRSF13B, COPS3, RASD1, PEMT* |
| 17q | q22 | 55,950,001 | q22 | 57,384,000 | 1,434,000 | Deletion | **57.6** | 22.2 | **0.02** | 0.27 | *RNF43, VEZF1, SEPT4, TEX14, RAD51C, PPM1E, TRIM37, SKA2* |
| 17q | q24.1 | 62,839,470 | q24.1 | 63,914,355 | 1,074,886 | Deletion | **51.5** | 22.2 | 0.07 | 0.49 | *AXIN2, GNA13* |
| 17q | q24.3 | 68,762,000 | q24.3 | 70,569,000 | 1,807,001 | Deletion | **57.6** | 27.8 | **0.02** | 0.27 | *SOX9* |
| 17q | q25.1 | 70,650,001 | q25.1 | 71,431,000 | 781,000 | Deletion | 54.5 | 22.2 | **0.04** | 0.38 | *SDK2, SSTR2, CDC42EP4* |
| 17q | q21.2 | 38,808,001 | q21.2 | 39,765,000 | 957,000 | Deletion | 45.5 | 27.8 | 0.25 | 0.84 | *KRT10, KRTAP, KRT23, KRT19, KRT13, KRT15* |
| 18q | q21.1 | 44,700,000 | q21.1 | 45,757,000 | 1,057,000 | Deletion | 42.4 | 72.2 | 0.08 | 0.49 | *SMAD2* |
| 18q | q21.1 | 47,205,001 | q21.2 | 52,112,000 | 4,907,000 | Deletion | 48.5 | 72.2 | 0.14 | 0.63 | *SMAD4, MEX3C, MAPK4, MBD1, DCC* |
| 18q | q21.32 | 57,580,001 | q21.32 | 58,096,000 | 516,000 | Deletion | 42.4 | 72.2 | 0.08 | 0.49 | *MC4R* |
| 18q | q22.1 | 64,820,001 | q22.1 | 66,314,000 | 1,494,000 | Deletion | 48.5 | 72.2 | 0.14 | 0.63 | *-* |
| 19p | p12 | 23,644,001 | p12 | 3,911,000 | 267,000 | Deletion | 27.3 | 22.2 | 0.75 | 0.94 | *-* |
| 19q | q11 | 27,977,001 | q11 | 28,915,000 | 938,000 | Deletion | 27.3 | 16.7 | 0.50 | 0.90 | *-* |
| 20p | p12.1 | 14,580,001 | p12.1 | 15,645,000 | 1,065,000 | Deletion | 21.2 | 38.9 | 0.20 | 0.79 | *MACROD2* |
| 21q | q11.2 | 15,306,001 | q21.1 | 18,048,000 | 2,742,000 | Deletion | 30.3 | 38.9 | 0.55 | 0.90 | *LIPI, ABCC13, SAMSN1, NRIP1, USP25* |
| 21q | q21.2 | 25,912,001 | q21.3 | 26,521,000 | 609,000 | Deletion | 39.4 | 33.3 | 0.77 | 0.94 | *-* |
| 21q | q22.11 | 32,086,001 | q22.11 | 33,234,000 | 1,148,000 | Deletion | 39.4 | 33.3 | 0.77 | 0.94 | *TIAM1, SOD1* |
| 21q | q21.3 | 30,116,001 | q21.3 | 30,916,000 | 800,000 | Deletion | 36.4 | 33.3 | 1.0 | 1.00 | *USP16, BACH1, GRIK1* |
| 21q | q22.3 | 43,545,000 | q22.3 | 46,807,000 | 3,262,001 | Deletion | 36.4 | 33.3 | 1.0 | 1.00 | *ABCG1, TMPRSS3, TFF1, PDE9A, PKNOX1, CBS, SIK1, RRP1B, PDXK, AGPAT3, ICOSLG, DNMT3L, TRPM2, SUMO3, PTTG1IP, ITGB2, ADARB1* |
| 22q | q12.1 | 28,682,001 | q12.2 | 29,677,000 | 995,000 | Deletion | 51.5 | 55.6 | 1.0 | 1.00 | *ZNRF3, CHEK2, XBP1, KREMEN1, EWSR1* |
| 22q | q11.1 | 17,573,001 | q11.21 | 18,619,000 | 1,046,000 | Deletion | 45.5 | 55.6 | 0.57 | 0.91 | *CECR1, BCL2L13, BID, TUBA8* |
| 22q | q13.2 | 43,027,001 | q13.2 | 43,310,000 | 283,000 | Deletion | 48.5 | 55.6 | 0.77 | 0.94 | *CYB5R3, ARFGAP3, PACSIN2* |

B) Amplification MCRs within MSS cohorts

| **Chr Arm** | **Chr band start** | **Start position (bp)** | **Chr band end** | **End position (bp)** | **Length MCR (bp)** | **Type of CNA** | **% of *BRAF*mut/ MSS cancers affected**  **(n=33)** | **% of *BRAF*wt/ MSS cancers affected (n=18)** | **p Value** | **Adjusted p value** | **Potential Cancer Related Genes Involved** |
| --- | --- | --- | --- | --- | --- | --- | --- | --- | --- | --- | --- |
| 5p | p14.3 | 22,911,001 | p14.1 | 25,650,000 | 2,739,000 | Amp | 15.2 | 22.2 | 0.70 | 0.730 | *PRDM9, CDH10* |
| 5p | p13.2 | 37,341,001 | p13.2 | 40,316,000 | 2,975,000 | Amp | 12.1 | 22.2 | 0.43 | 0.484 | *GDNF, EGFLAM, LIFR, OSMR, RICTOR, DAB2* |
| 7p | p21.3 | 7,492,001 | p21.1 | 20,814,000 | 13,322,000 | Amp | 9.1 | **38.9** | **0.02** | **0.051** | *PHF14, ARF4L, ETV1, AGR2/3, BZW2, HDAC9, TWIST1, MACC1, ITGB8, ABCB5* |
| 7q | q21.11 | 81,993,001 | q21.11 | 82,532,000 | 539,000 | Amp | 3.0 | **38.9** | **0.002** | **0.012** | *CACNA2D3, PCLO* |
| 7q | q36.2 | 154,436,001 | q36.3 | 159,119,000 | 4,683,000 | Amp | 3.0 | **38.9** | **0.002** | **0.012** | *DPP6, INSIG1, SHH, RNF32, MNX1, LMBR1, PTPRN2, NCAPG2, VIPR2* |
| 8q | q24.21 | 128,085,001 | q24.21 | 129,127,000 | 1,042,000 | Amp | **48.5** | 16.7 | **0.035** | 0.079 | *MYC, PVT1* |
| 8q | q23.1 | 109,055,001 | q23.2 | 109,888,000 | 833,000 | Amp | 45.5 | 16.7 | 0.065 | 0.097 | *RSP02, EIF3E* |
| 8q | q24.11 | 117,636,001 | q24.11 | 118,097,000 | 461,000 | Amp | 45.5 | 16.7 | 0.065 | 0.097 | *EIF3H, UTP23, RAD21* |
| 8q | q24.22 | 132,704,000 | q24.22 | 135,106,000 | 2,402,001 | Amp | 45.5 | 16.7 | 0.065 | 0.097 | *EFR3A, PHF20L1, SLA, WISP1, NDRG1, ST3GAL1* |
| 8q | q22.3 | 100,850,001 | q22.3 | 102,660,000 | 1,810,000 | Amp | 42.4 | 16.7 | 0.07 | 0.097 | *VPS13B, RGS22, SPAG1, RNF19A,ANKRD46, SNX31, PABPC1, YWHAZ, ZNF706, GRHL2* |
| 13q | q14.11 | 40,387,001 | q14.11 | 44,439,000 | 4,052,000 | Amp | 12.1 | **66.7** | **0.0001** | **0.002** | *FOXO1, ELF1, DGKH, EPSTI7, ENOX1* |
| 13q | q21.33 | 70,105,001 | q21.33 | 70,950,000 | 845,000 | Amp | 21.2 | **61.1** | **0.007** | **0.030** | *KLHL1* |
| 18q | q11.2 | 19,636,000 | q11.2 | 20,838,000 | 1,202,001 | Amp | **21.2** | 0.0 | **p=0.04** | 0.080 | *GATA6, CTAGE, RBBP8, CABLES27* |
| 20p | p12.1 | 15,874,001 | p11.23 | 20,739,000 | 4,865,000 | Amp | 15.2 | 38.9 | 0.08 | 0.103 | *MACROD2, PCSK2, DSTN, SNX5, SEC23B, RIN2, RALGAPA2* |
| 20p | p11.21 | 24,267,001 | p11.1 | 25,184,000 | 917,000 | Amp | 9.1 | **44.4** | **p=0.01** | **0.030** | *ACSS1* |
| 20p | p12.3 | 5,219,001 | p12.3 | 5,750,000 | 531,000 | Amp | 18.2 | 33.3 | 0.30 | 0.360 | *PROKR2* |
| 20q | q11.23 | 35,001,000 | q11.23 | 36,050,000 | 1,049,001 | Amp | 24.2 | 38.9 | 0.73 | 0.73 | *TGIF2, NDRG3, DSN1, SAMHD1, RPN2, GHRH, SRC* |
| 20q | q13.2 | 50,823,001 | q13.33 | 56,496,000 | 5,673,000 | Amp | 18.2 | **55.6** | **p=0.01** | **0.03** | *AURKA, TSHZ2, BCAS1, PFDN4, CASS4, BMP7, CTCFL, RAE1, ZBP1, CTCFL, PMEPA1* |

1. MCRs affecting ≥10% *BRAF*mut/MSI cohort

| **Chr Arm** | **Chr band start** | **Start position (bp)** | **Chr band end** | **End position (bp)** | **Length MCR (bp)** | **Type of CNA** | **% of *BRAF*mut/MSI cancers affected**  **(n=30)** | **Potential Cancer Related Genes Involved** |
| --- | --- | --- | --- | --- | --- | --- | --- | --- |
| 2q | q37.3 | 240,832,001 | q37.3 | 242,518,000 | 1,686,000 | Deletion | 13.3 | *GPC1, CAPN10, KIF1A, SEPT2, STK25* |
| 3p | p14.2 | 60,037,001 | p14.2 | 61,178,000 | 1,141,000 | Deletion | 33.3 | *FHIT* |
| 4q | q22.1 | 91,210,000 | q22.1 | 92,140,000 | 930,001 | Deletion | 10.0 | *FAM190A* |
| 16p | p13.3 | 5,831,001 | p13.3 | 7,534,000 | 1,703,000 | Deletion | 46.7 | *RBFOX1* |
| 16q | q23.1 | 78,106,000 | q23.2 | 79,335,000 | 1,229,001 | Deletion | 16.7 | *WWOX* |
| 17q | q22 | 55,950,001 | q22 | 57,384,000 | 1,434,000 | Deletion | 13.3 | *RNF43, VEZF1, SEPT4, TEX14, RAD51C, PPM1E, TRIM37, SKA2RNF43* |
| 17q | q24.3 | 68,762,000 | q24.3 | 70,569,000 | 1,807,001 | Deletion | 10.0 | *SOX9* |
| 20p | p12.1 | 14,580,001 | p12.1 | 15,645,000 | 1,065,000 | Deletion | 20.0 | *MACROD2* |
| 21q | q22.11 | 32,086,001 | q22.11 | 33,234,000 | 1,148,000 | Deletion | 10.0 | *TIAM1, SOD1* |
| 7p | p21.3 | 7,492,001 | p21.1 | 20,814,000 | 13,322,000 | Amplification | 10.0 | *PHF14, ARF4L, ETV1, AGR2/3, BZW2, HDAC9, TWIST1, MACC1, ITGB8, ABCB5* |
| 7q | q11.23 | 76,342,000 | q21.23 | 76,615,000 | 273,000 | Amplification | 13.3 | *-* |
| 7q | q36.2 | 154,436,001 | q36.3 | 159,119,000 | 4,683,000 | Amplification | 13.3 | *DPP6, INSIG1, SHH, RNF32, MNX1,LMBR1, PTPRN2, NCAPG2, VIPR2* |
| 8p | p11.21 | 40,221,000 | p11.21 | 42,013,000 | 1,792,000 | Amplification | 10.0 | *ZMAT4, SFRP1, GOLGA7, GINS4, NKX6-3, ANK1, KAT6A* |
| 13q | q14.11 | 40,387,001 | q14.11 | 44,439,000 | 4,052,000 | Amplification | 20.0 | *FOXO1, ELF1, DGKH, EPSTI7, ENOX1* |

Supporting Information - References

1. Nancarrow DJ, Handoko HY, Smithers BM, Gotley DC, Drew PA, Watson DI, et al. Genome-wide copy number analysis in esophageal adenocarcinoma using high-density single-nucleotide polymorphism arrays. Cancer Res. 2008 Jun 1;68(11):4163-72.

2. Dulak AM, Schumacher SE, van Lieshout J, Imamura Y, Fox C, Shim B, et al. Gastrointestinal adenocarcinomas of the esophagus, stomach, and colon exhibit distinct patterns of genome instability and oncogenesis. Cancer Res. 2012 Sep 1;72(17):4383-93.

3. Bond CE, Umapathy A, Ramsnes I, Greco SA, Zhen Zhao Z, Mallitt KA, et al. p53 mutation is common in microsatellite stable, BRAF mutant colorectal cancers. Int J Cancer. 2012 Apr 1;130(7):1567-76.

4. Bond CE, Umapathy A, Buttenshaw RL, Wockner L, Leggett BA, Whitehall VL. Chromosomal instability in BRAF mutant, microsatellite stable colorectal cancers. PLoS One. 2012;7(10):e47483.
